# Potential Activity of Non-Platinum Metal-Based Organic Complexes Against Different Cancer Cell Types

**DOI:** 10.3390/ph19060925

**Published:** 2026-06-12

**Authors:** Dobrina Tsvetkova, Stefka Ivanova, Danka Obreshkova

**Affiliations:** 1 Department of Pharmaceutical Chemistry, Faculty of Pharmacy, Medical University-Sofia, Dunavstr. 2, 1000 Sofia, Bulgaria; 2 Bulgarian Pharmaceutical Society, 1000 Sofia, Bulgaria; ivanovastefka_pharm@yahoo.com; 3 Multi-Profile Emergency Department, UMHATEM “N.I. Pirogov”, 1606 Sofia, Bulgaria; phddanka@yahoo.com

**Keywords:** non-platinum metal–organic complexes, anticancer activity, selectivity, toxicity, resistance

## Abstract

The disadvantages of Cisplatin in anticancer treatment are connected to its poor selectivity, resistance developed of cancers to the drug, and its toxicity against normal organs. An important strategy in anticancer treatment is the synthesis and clinical investigation of non-platinum metal complexes with superior anticancer activity and improved selectivity compared to Cisplatin, combined with lower toxicity, fewer side effects and decreased resistance of cancer to the drug. In the current study, we aim to summarize the potential of important non-platinum metal-based organic compounds as therapeutic agents against different cancer cell types. The review covers the general principles of chemotherapy. A literature analysis shows that organic complexes of the metalloids arsenic (As), boron (B), antimony (Sb), and selenium (Se), and of metals, such as Ag, Au, Co, Cu, Fe, Mn, Mo, Ni, Zn, Ce, Ga, Gd, Ir, Os, Pd, Re, Rh, Ru, Ti, and V, have been investigated for potential applications in cancer therapy. This is due to their antiproliferative effects against different cancer types: lung [Cd(II), Co(II), Cu(II), Ni(II), Mn(II), Ru(II), Zn(II)]; breast [Ag(I), Cu(I), Cu(II), Ir(III), Ni(II), Mn(II),. Rh(III), Ru(II)]; gastric [Cu(II), Cu(II)-La(III)]; colon [Ag(I), Cu(II), Ir(III), Pd(II), Rh(III), Ru(II), vanadium(V)]; colorectal [Ag(I), Co(II), Cu(II), Zn(II)]; liver [Ag(I), Co(II), Cu(II), Gd(III), vanadium(V)]; pancreatic [vanadium(IV)]; bladder [Ag(I), Cu(II), Ru(II)]; cervical [Ag(I), Au(I), Cu(I), Cu(II), Fe(II), Ir(III), Rh(III), Ru(II)]; testicular [vanadium(IV)]; prostate [Cu(II), Pd(II), Zn(II)]; leukemia [Ag(I), Co(II), Cu(II), Pd(II), Zn(II)]; sarcoma [Co(II), Ni(II), Zn(II)]; mesothelioma [Cu(II)]; neuroblastoma [Cu(II)]; glioma [Cu(II)]; and melanoma [Au(I), Cu(II), Pd(II), Ru(II)]. The main goals for increasing anticancer metal-based complexes include increasing anticancer activity and selectivity, reducing toxicity, and avoiding cancer cell resistance. Compared to Cisplatin, organocomplexes of copper, ferrocene, and ruthenium are more active. Ruthenium and copper complexes, in particular, are also more selective. Notably, ruthenium and ferrocene derivatives are less toxic than Cisplatin. Lastly, cancers appear to exhibit less resistance against copper, gold, ruthenium, palladium, and ferrocene complexes.

## 1. Introduction

Malignant cancers, of which there are over 100 different types, are the leading cause of lethality worldwide [1].

Non-platinum metal-based organic complexes have been found to exert a potential antiproliferative effect against different cancer cell types: lung, breast, gastric, colon, colorectal, liver, pancreatic, bladder, cervical, testicular, prostate, leukemia, sarcoma, mesothelioma, neuroblastoma, brain glioma, and melanoma [1]. Risk factors for cancer development are important to know, as this can contribute to more effective prediction and increased potential for the prevention of cancer cases among the population. Cancer is the second leading cause of death in humans after cardio- and cerebrovascular diseases, and its associated death rate is the highest among all major diseases [1]. Due to this reason, more important strategies must be directed to the development of more effective anticancer drugs. Chemotherapy is associated with developed resistance and drug toxicity. The disadvantages of Cisplatin in anticancer treatment are connected to its poor selectivity, resistance developed of cancers to the drug, and its toxicity against normal organs [1]. This is the reason for the development of a new trend in anticancer therapy, which is focused on searching for more active organocomplexes. The aim of the study is focused on summarizing the potential of important non-platinum metal-based organic compounds as therapeutic agents against different cancer cell types. These data can contribute to further searching and choosing of the more active organocomplexes with decreased resistance and toxicity as appropriate anticancer agents.

Risk factors for cancer development are also shown, as they are important to know, since this can contribute to more effective prediction and increased potential for the prevention of cancer cases among the population. Principles and therapeutic strategies in cancer chemotherapy are presented in order to express the connection between the correct therapy, employing short-term and intensive treatment courses, and the decrease in the development of drug resistance.

## 2. Risk Factors for Cancer Development

Factors contributing to cancer development can be genetic and environmental. At the molecular level, various genetic tumor-specific mutations can occur in somatic cell DNA, which defines the biological features of neoplastic transformation, characterized as uncontrolled cell proliferation, local tissue invasion, and distant metastases. Tumor-specific mutations in somatic cells lead to uncontrolled cell growth (Table 1). Initiation is characterized by genetic alterations that lead to the dysregulation of biochemical signaling pathways such as Phosphoinositide 3-kinase (PI3K)/Protein Kinase B (AKT), connected with unregulated growth, and Mitogen-Activated Protein Kinase (MAPK)/Extracellular Signal-Regulated Kinase (ERK), the dysregulation of which increases the cells’ continuous proliferative state [1].

During the promotion stage, the accumulation of actively proliferating preneoplastic cells is observed. Progression is characterized by fast tumor growth and represents the phase between a premalignant lesion and the development of invasive cancer. Metastasis is the spread of cancer cells from the primary site to other parts of the body through the bloodstream or the lymph system [1].

### 2.1. Genetic Risk Factors for Cancer Development

The inactivation of tumor suppressor genes (p53, RAS), and dysregulation of positive and negative proliferation signals are the main reasons for the transformation of a typical cell into a malignant one [2].

Genetic abnormalities can affect different genes, such as p53 [3], VHL, WT1 [4], BRCA1 [5], APC [6], XP [7], NF1, NF2, RET [8], which are summarized in Table 1.

**Table 1:** Genetic risk factors for the development of cancer.

Cell Cycle Genes	DNA Repair Genes	Signal Transduction Genes	Tissue Organization Genes
p53 [3] (brain and breast cancer)	BRCA1, BRCA2 (breast and ovarian cancer) [5]	MET (papillary renal cell carcinoma)	APC (colon cancer) [6]
p16 (melanoma), Rb1 (retinoblastoma)	MLH1, MSH2, MSH6, PMS1, PMS2 (colorectal cancer) [6]	NF1 (neurofibroma) [8]	E-cadherin (gastric cancer) [8]
VHL (renal cancer) [4], WT1 (Wilms cancer) [4]	XPA, XPB, XPC, XPD, XPF, XPG (skin cancer) [7]	RET (adrenal pheochromocytoma) [8]	NF2 (neurinoma) [8]

The development of cancer depends on ribosome biogenesis [9] and is associated with mutations in genes responsible for the synthesis of ribosomal proteins [10], which play an important role in the regulation of cell proliferation [11]. The increase in ribosome biogenesis leads to carcinogenesis through the down-regulation of cell tumor suppressor potential [12]. In the process of cancer development, several onco-ribosomes are expressed in cancer cells [13]. A mutation in the ribosomal protein RPL10 is the factor that leads to the development of pediatric T-cell acute lymphoblastic leukemia (T-ALL). Mutations in the ribosomal proteins RPS15 and uS19 lead to chronic lymphocytic leukemia (CLL). Endometrial cancer is associated with changes in RPL22 and familial colorectal cancer is linked to RPS20. The reasons for the development of T-cell acute lymphoblastic leukemia (T-ALL) and of glioblastomas are mutations in ribosomal proteins RPL5 and RPL11 [12,13].

DNA damage can also be caused by substances produced in the body. Macrophages and neutrophils in inflamed epithelium initiate colonic tumorigenesis due to deoxyribonucleic acid (DNA) damage caused by the overproduction of free radicals such as reactive oxygen and nitrogen species [14], which are associated with cancer pathogenesis [15]. Additionally, under a high-fat diet, high levels of bile acids cause DNA damage and contribute to the development of colon cancer.

### 2.2. Physical, Chemical, and Biological Risk Factors for Cancer Development

DNA damage can arise from exposure to exogenous physical, chemical, and biological factors and carcinogens in the environment [1]. Heterocyclic amines are important carcinogenic compounds, which can form in overcooked meat and fish when amino acids and creatine react at high temperatures. Heterocyclic amines are transformed into carcinogens through a mechanism involving their oxidation in the body to N-hydroxy derivatives, mainly by cytochrome P_450_ CYP1A2 enzymes in the liver. Then, acetyltransferases and sulfotransferases activate N-hydroxy derivatives into highly reactive electrophiles, which form DNA adducts by binding covalently to DNA bases (especially guanine). These adducts cause DNA mutations, leading to uncontrolled cell growth and tumor formation [16].

Physical, chemical, and biological risk factors contributing to cancer development are presented in Table 2.

## 3. Principles and Therapeutic Strategies in Cancer Chemotherapy

The treatment of malignant tumors includes therapy, control of the malignant process, and palliative care [29]. Methods are complex and include surgical treatment [30] and chemo-, immuno-, hormone, and radiation therapy. Drug control refers to stopping the growth and spread of cancer. Palliative strategies are employed in the advanced stages of cancer with metastases. About 70% of patients that are operated on develop distant metastases at varying durations after local treatment, which then necessitates systemic application of chemotherapeutics [29].

Chemotherapy is the use of drugs for the treatment of malignant diseases, which is a component of the complex therapy of malignant tumors and has cytoreductive therapeutic aims, as follows:

1. Decreasing the number of tumor cells and the size of the local cancers;
2. Reducing or eliminating migrating tumor cells in the body or inhibiting cancer metastasis [29].

The indications for chemotherapy include the following [4]:

1. Cases where operative removal is possible;
2. As an additional adjuvant method to attack the spread of micrometastases after surgery or radiation therapy;
3. Through the application of cytostatics before a surgical procedure to reduce the size of the tumor through non-adjuvant therapy;
4. As maintenance chemotherapy at low doses after cycles have been completed to prolong the period of achieved remission;
5. Self-administered chemotherapy for advanced tumors and hematological neoplasms.

The principles and therapeutic strategies in chemotherapy are as follows [29]:

1. Selective toxicity against cancer cells without affecting normal tissues and organs;
2. Administered in repeated cycles according to a scheme, which is based on the proliferative features of the cells and the restoration of hematopoiesis;
3. Personalization of therapy for tumors that have proven target markers and require target-oriented treatment;
4. Therapeutic monitoring of damage to hematopoietic cells and other rapidly proliferating cells;
5. Intensification of treatment with high-dose chemotherapy in the shortest possible interval in highly sensitive tumors or regional chemotherapy by local administration of cytostatics, but not in patients with impaired liver function and those undergoing irradiation of the abdominal area.

Challenges associated with chemotherapy include resistance and toxicity. Resistance varies with different tumors, with a response being difficult to achieve in some (e.g., melanoma, lung, adrenal), and others become resistant during treatment cycles, especially in cases where a suboptimal dose is administered. The development of drug resistance is minimized through employing short-term and intensive treatment courses. Multiple-drug resistance can arise due to the genetically encoded transmembrane P-glycoprotein [31]. Anticancer drugs lack selectivity for tumor cells, and can also kill normal cells with high proliferative activity, for example, hematopoietic bone marrow and gastrointestinal tract cells. The use of drugs for the treatment of malignant tumors is associated with adverse reactions (nausea, vomiting, stomatitis, bone marrow depression, and alopecia), the monitoring and control of which are mandatory to ensure accurate dosing and safe chemotherapy [32].

Based on theoretical knowledge about the development of tumors and their molecular–biological features, the main areas that are being investigated for the discovery of new antitumor drugs are outlined as follows [33]:

1. The targets of antitumor drugs—DNA, purines, or pyrimidines, which are needed as precursors for the synthesis of DNA and RNA;
2. The influence of neoangiogenesis in solid tumors, which inhibits their growth (antiangiogenic drugs);
3. The discovery of new non-platinum metal-based complexes.

## 4. Approved Metal-Based Complexes as Drugs Beyond Cancer Treatment

**Figure 1:**
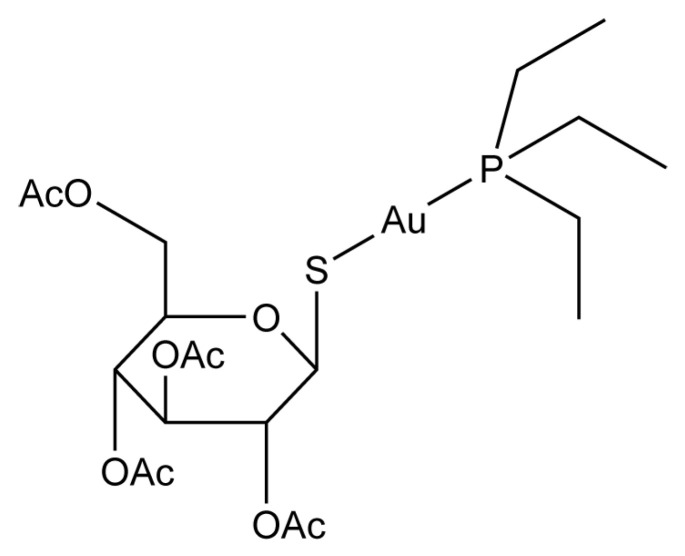
Chemical structure of Auranofin.

Auranofin (Figure 1) is an approved drug–metal complex.

Orally active Auranofin (Figure 1) was approved by the FDA in 1985 as an antirheumatic drug that inhibits cathepsins. The drug was studied in clinical trials for its activity towards chronic lymphocytic leukemia, non-small-cell or small-cell lung cancer, and ovarian cancer [34].

It has been reported that the mechanism of action of Auranofin for the suppression of proliferation of Calu-6, A549, SK-LU-1, NCI-H460, and NCI-H1299 lung cancer cells is connected to the drug’s ability to stimulate increased production of reactive oxygen species (ROS) and decrease glutathione (GSH) concentration in cancer cells. These findings support the correlation between the oxidative stress induced by Auranofin and the resulting cancer cell death [35].

It is known that thioredoxin reductase (TrxR) protects cells from oxidative stress. In many cancer cells, the concentration of thioredoxin reductase is higher than in non-cancer cells, which is explained by cancer cells attempting to enhance their stability against the effects of reactive radicals. It is reported that Auranofin acts as a pro-oxidant agent, which is evidenced by the fact that the compound changes the balance between cell oxidation and reduction systems [32,34]. This mechanism of activity involves the blockage of the anti-oxidative system by thioredoxin reductase and results in increased reactive oxygen species, which contribute to cell apoptosis [35].

A specific advantage of Auranofin over Cisplatin lies in that it can overcome platinum resistance in ovarian cancer by inducing cell death in the ovarian cancer cell line OV2008/C13. This effect is due to the inhibition of thioredoxin reductase [36]. The same mechanism has been reported for the anticancer activity of this compound against glio- and neuroblastoma brain tumors [37].

It is known that the proteasome is a specific protein complex that plays an important role in cancer cell growth progression by regulating protein cell homeostasis, specifically through degrading damaged proteins. Auranofin has been found to exert its cytotoxic effect by inhibiting proteasome-associated deubiquitinases (DUBs). This mechanism is involved in the induced apoptosis of HepG2 liver hepatocellular carcinoma cells and in the suppression of tumor growth in MCF-7 breast cancer cells, chronic myeloid leukemia (CML), and prostate cancer [38].

The main pharmacological mechanisms of Auranofin’s anticancer effects are summarized in Table 3.

**Figure 2:**
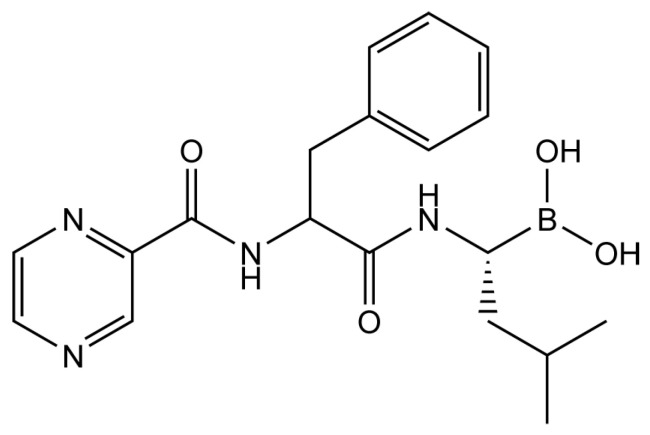
Chemical structure of Bortezomib.

The US approved Bortezomib (Figure 2) for multiple myeloma treatment in 2003. As the first Food and Drug Administration (FDA)-approved 26S proteasome inhibitor, Bortezomib has been used to treat non-small-cell lung, breast, pancreatic, renal, ovarian, and prostate cancers, as well as lymphoma and melanoma [39].

The mechanisms of Bortezomib’s anticancer effects include the following [39]:

1. Inhibition of 26S proteasome by binding the catalytic site of the 26S proteasome via Bortezomib’s boron atom;
2. Suppression of tumor cell proliferation by decreasing cyclin D and interleukin 6 transcription;
3. Activation of tumor cell apoptosis through the release of mitochondrial cytochrome C;
4. Increased caspase activity.

## 5. Antiproliferative Activity of Non-Platinum Metal-Based Organic Complexes Against Different Cancer Types

After cardio- and cerebrovascular diseases, cancer is the second leading cause of death in humans, and its associated death rate is the highest among all major diseases. Every year, cancer is responsible for more than 13% of all deaths globally. Lung, breast, gastric, colorectal, and prostate cancers are the most common types; notably, the rates of breast carcinomas in women, and lung, prostate, and colon carcinomas in men have recently increased. The most common cancer types are as follows [40].

1. Carcinomas: Conjunctival, oral, nasopharyngeal, head and neck, lung, breast, gastric, colorectal, hepatocellular, bladder, ovarian, cervical, testicular, and prostate.
2. Sarcomas: Osteosarcoma and rhabdomyosarcoma.
3. Blastomas: Neuro-, retino-, and hepatoblastoma.
4. Lymphomas: Non-Hodgkin lymphoma and peripheral T-cell lymphoma.
5. Multiple myeloma.
6. Mesothelioma.
7. Melanoma.

Complexes of the metals listed in Table 4 represent potential therapeutic agents for medical applications due to their antiproliferative activity [41].

### 5.1. Human Laryngeal Carcinoma

**Figure 3:**
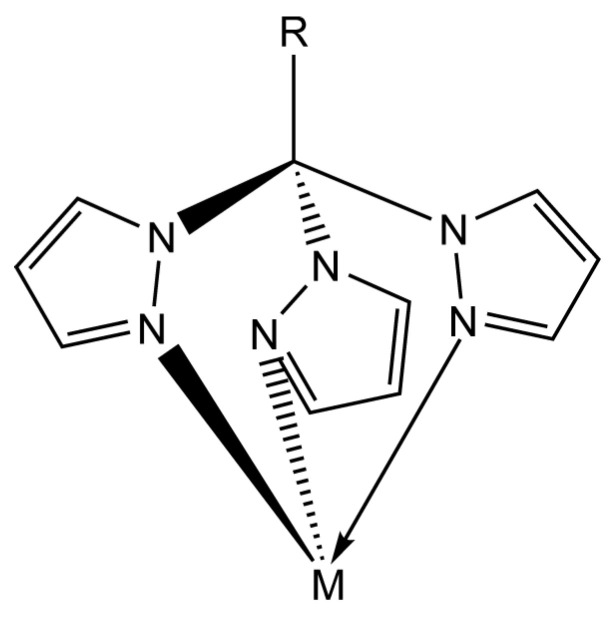
Chemical structure of scorpionates. (“M” is metal).

The coordination compounds scorpionates (Figure 3)—formed by tridentate ligands that bind the metal with two in-plane donors—are promising anticancer agents. Tridentate chelating ligands play an important role in organometallic chemistry because they allow the formation of stable metal complexes. Scorpionates were discovered by Trofimenko in 1960, with the term scorpionate referring to the third donor site, formed by the metal and the other two donor atoms, which reaches over the plane, resulting in a scorpion-like conformation. It has been found that complexes with scorpionate ligands, derived from bis- or tris-(pyrazol-1-yl)-borate moieties, show anticancer properties [64].

Various studies have investigated the in vitro antiproliferative potential of scorpionates of Ag(I) [65], Cu(I) [66,67], Co(II) [68], Mn(II) [69], Zn(II) [68], iron, and vanadium [67]. Notably, Ag(I) scorpionates with tris(2-pyridyl)phosphine oxide have been described to exhibit cytotoxic activity against the human laryngeal carcinoma Hep-2 cell line [65].

### 5.2. Lung Cancer

Lung cancer is a major neoplastic disease and is related to 90% of all cancer-related deaths globally. About 54% of malignant tumors are metastatic lung lesions [70]. In 2020, 2.2 million people were reported to have lung cancer and 1.8 million deaths were caused by it. The highest incidence rates of lung cancer have been reported among men in Central and Eastern Europe but, among women, the highest incidence rates have been described in North America and Northern Europe [40].

About 98–99% of all lung cancers are carcinomas, malignant tumors characterized by uncontrolled epithelial cell growth. The three main subtypes of non-small-cell lung carcinoma are adenocarcinoma (40%), squamous cell carcinoma (30%), and large-cell carcinoma (15%). In women, the incidence of lung cancer has doubled since 1990 [71]. Today, adenocarcinoma is the most common lung cancer subtype in humans worldwide [72].

Rare subtypes of lung cancer include pulmonary enteric adenocarcinoma, bronchioloalveolar carcinoma, adenosquamous carcinoma, bronchial gland carcinoma, sarcomatoid carcinoma, and carcinoid tumors. Lung sarcomas are caused by the malignant transformation of connective tissues (nerve, muscle, and bone), which arise from mesenchymal cells. Lymphomas and melanomas (from lymphoid and melanocyte cell lineages) are rare types of lung cancer [73].

Complexes of Cd(II) Co(II), Cu(II), Ni(II), and Mn(II) with the Schiff base ligand 4-((Z)-(4-nitrophenylimino)methyl)benzene-1,3-diol, derived from the condensation of 2,4-dihydroxybenzaldehyde and p-nitroaniline, have been screened for their antiproliferative properties. For example, it has been found that the Cd(II) complex shows higher cytotoxic activity against human lung cancer cell lines [74], and the copper complex of salicylaldehyde pyrazole hydrazone induces apoptosis by up-regulating integrin β4 in H322 lung carcinoma cells [75].

It has been reported that the mechanism of action of half-sandwich iridium and cyclometallic iridium compounds is based on the induction of cancer cell apoptosis due to mitochondria, lysosome, and endoplasmic reticulum damage caused by oxidative stress, as a result of these agents stimulating the increased production of reactive oxygen species in cancer cells [76]. Important advantages of these iridium (III) complexes include their low toxicity against normal cells and the ease of their entry into lung tumor cells [76].

It has been proven that cationic NHC copper(I) complexes, coordinated to 2,2′-bis-pyridyl ligands [77], and arene Ru(II) agents, containing 1,2,3,4-tetrahydroisoquinoline amino alcohol ligands, display anticancer properties against the human lung carcinoma A549cell line [78]. The pro-apoptotic potential of the of 6-methoxyquinoline compounds of Cu(II) and Zn(II) [79] and of pyridoxal–vanadium(IV) towards the lung A549 cell line is a result of increased intracellular oxidative stress [80].

It has been suggested that Co(II) and Cu(II) salicylaldehyde Schiff base derivatives [81] and Ni(II) oxyquinoline–bipyridine compounds [82] suppress growth in human lung adenocarcinoma A549/DDP cells.

It has been shown that photoactive polypyridyl Ru(II) derivatives exhibit cytotoxic activity against A549 non-small-cell lung cancer and MDA-MB-231 triple-negative breast cancer cells [83] with the following ligands:

1. 2,2′-bipyridine/6,6′-dimethyl-2,2′-bipyridine;
2. 1,10-phenanthroline/6,6′-di-methyl-2,2′-bipyridine;
3. 4,7-diphenyl-1,10-phenanthroline)/6,6′-dimethyl-2,2′-bipyridine;
4. bathophenanthrolinedisulfonate, 6,6′-dimethyl-2,2′-bipyridine.

**Figure 4:**
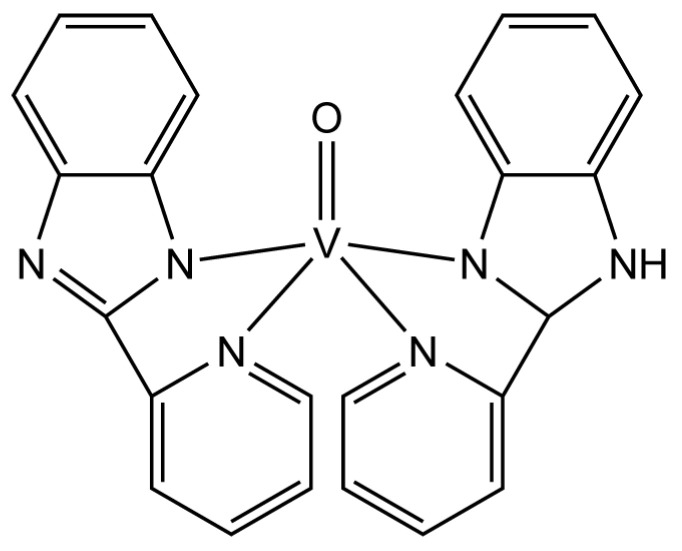
Chemical structure of vanadium(V)-pyridylbenzimidazole complex.

The DNA binding activity of the vanadium(V)-pyridylbenzimidazole complex (Figure 4), which is active against lung cancer cell line A549 [84], has been reported.

### 5.3. Breast Cancer

Breast cancer is one of the leading causes of cancer in both sexes (11.6%), but is 100 times more common in females than in males [85]. In 2020, it was estimated that breast cancer was responsible for 11.7% of all cancer cases. Worldwide, breast cancer is the most commonly diagnosed cancer among women, accounting for 25% of all cases [86]. Developed countries, including Australia, New Zealand, Europe, and Northern America, have the highest incidence rates [85]. The disease is the fifth leading cause of mortality, accounting for around 460 million deaths each year and 6.9% of all cancer deaths in females [87].

There are more than 18 subtypes of breast cancer, such as ductal and lobular carcinoma [85]. In breast cancer, the major contributor to high mortality is tumor metastasis, caused by the presence of cancer stem cells, for which treatments employing different metal complexes—including cobalt, nickel, copper, zinc, manganese, iridium osmium, palladium, and ruthenium—have been developed [88].

**Figure 5:**
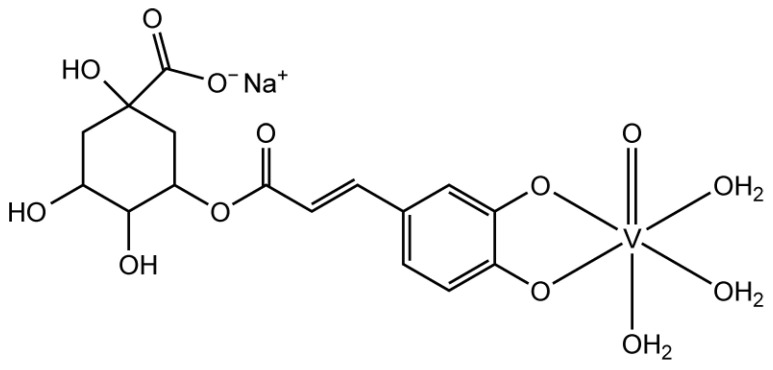
Chemical structure of oxidovanadium(IV) complex of chlorogenic acid.

An oxidovanadium(IV) complex with 3-(3,4-dihydroxycinnamoyl)quinic acid (Figure 5) exhibits cytotoxicity against the human breast cancer cell line SKBR3 [89].

**Figure 6:**
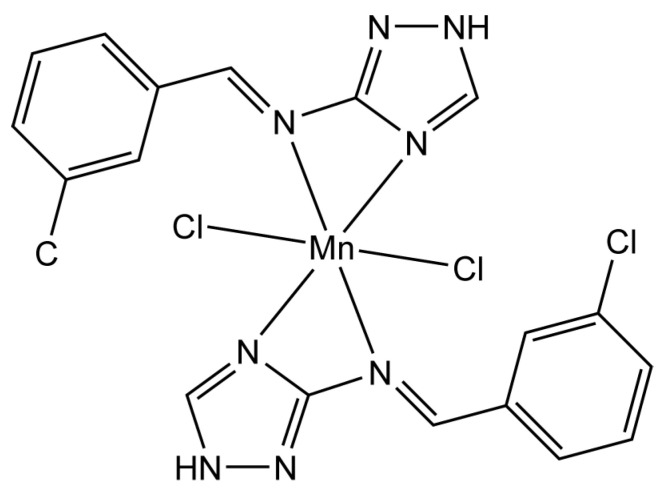
Chemical structure of Mn(II) complex with N-(-(3-chlorobenzylidene)-1H-1,2,4-triazol-3-amine.

It has been shown that the following metal-based complexes exert anticancer properties against the human breast cancer MCF7 cell line:

1. Dinuclear Cu(I) complexes, containing cyclodiphosphazane derivatives and a pyridyl ligand [90];
2. Cationic NHC Cu(I) compounds coordinated to 2,2′-bis-pyridyl ligands [77];
3. Cu(II)-tropolone derivative [91];
4. Cu(II)-N,N,O-chelating agents [92];
5. Cu(II) and Ni(II) complex Schiff base ligands, and 3-(2-(1-(1H-benzimidazol-2-yl)ethylidene)hydrazinyl)quinoxa-lin-2(1H)-one, synthesized by the condensation of 3-hydrazinylquinoxalin-2(1H)-one and 1-(1H-benzoimidazol-2-yl) ethanone [93];
6. Arene Ru(II) compounds containing 1,2,3,4-tetrahydroisoquinoline amino alcohol ligands [78];
7. Vanadium(V)-pyridylbenzimidazole complex [84];
8. Mn(II) complexes with N-(3-chlorobenzylidene)-1H-1,2,4-triazol-3-amine (Figure 6), and N-(4-methoxybenzylidene)-1H-1,2,4-triazol-3-amine, with 4-methoxybenzylidene on the 3-chlorobenzylidene [94].

Agents with cytotoxicity against human breast carcinoma MDA-MB-231 cells include the following:

1. Polyoxometalate-based complexes of Ag(I) with 4-(5-(1H-imidazol-2-yl)-1H-1,2,4-triazol3-yl)pyridine);
2. Di-*n*-butyltin(IV) [N-(3-ethoxy-2-oxidobenzylidene)-N′-(oxidomethylene)hydrazine] with an IC_50_ value of 48.32 µg/mL [95];
3. Cu(II) and Ag(I) derivatives of 1,3-diaryltriazene-substituted sulfonamides [96];
4. Ru(II) agents with N,O-ligands [97] and Ru(II) iminophosphorane complexes [98];
5. Arene Ru(II) complexes, containing 1,2,3,4-tetrahydroisoquinoline amino alcohol ligands [78].

It has been reported that di-*n*-butyltin(IV) [N-(3-ethoxy-2-oxidobenzylidene)-N′-(oxidomethylene)hydrazine] has an IC_50_ value of 48.32 µg/mL [95].

Promising photoactive agents that induce reactive oxygen species production and apoptosis of MDA-MB-231 cells [84] include Ru(II) complexes with the following ligands [83]:

1. 2,2′-bipyridine/6,6′-dimethyl-2,2′-bipyridine;
2. 1,10-phenanthroline/6,6′-di-methyl-2,2′-bipyridine;
3. 4,7-diphenyl-1,10-phenanthroline)/6,6′-dimethyl-2,2′-bipyridine;
4. bathophenanthroline disulfonate-6,6′-dimethyl-2,2′-bipyridine.

Benzimidazole C,N-cyclometalated Ru(II), Ir(III), and Rh(III) derivatives [99] and Pd(II) complexes with a thiazoline ligand [100] also display antiproliferative activity.

**Figure 7:**
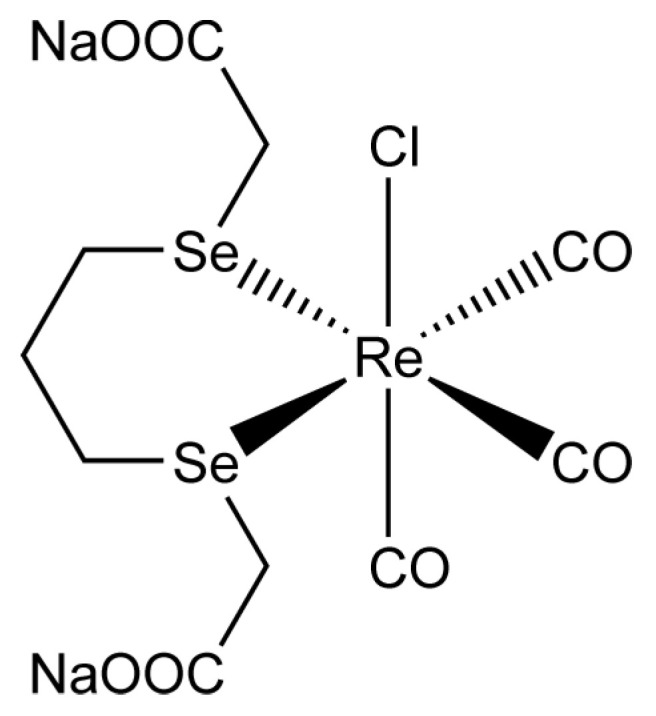
Chemical structure of Re(I) diselenoether complex.

The diselenoether Re(I) complex (Figure 7) is designed to combine the cytotoxic effect of rhenium with the antiproliferative activity of selenium. This agent is one of the few Re(I) compounds that has been evaluated for in vivo activity; it has been observed to inhibit tumor growth in MDA-MB-321 breast cancer in mice, with a 43% reduction in the tumor volume and minimal toxic side effects [101].

### 5.4. Gastric Cancer

Worldwide, gastric cancer has a high incidence and mortality [102]. Notably, copper Cu(II)-based coordination polymers [103] and the Cu(II)-La(III) thiophene-2,5-dicarboxylic acid/N,N′-dimethylacetamide complex have been proven to inhibit the proliferation of human gastric cancer cells [104].

### 5.5. Colon and Colorectal Cancer

Colorectal cancer is the third most common cancer overall worldwide, being the third most common in men and the second most common in women. The disease is the second most common cause of cancer-related death (9.2%) after lung cancer, showing an increase every year in terms of both morbidity and mortality. Over 50% of patients are diagnosed at or beyond stage III, when distant metastasis has already happened. The essential mechanism for inhibiting colon cancer is the suppression of cell proliferation and the induction of apoptosis [105].

Ag(I) and Cu(II) complexes of 1,3-diaryltriazene-substituted sulfonamides show cytotoxicity towards human colorectal adenocarcinoma (DLD-1) [96]. Cu(II)–phenanthroline agents also exhibit antitumor effects through inducing apoptosis of the colorectal cancer cell lines HT-29, LS174T, and Caco-2 as a result of stimulating the formation of reactive oxygen species (ROS) [106].

Potential anticancer effects against HT-29 colon cancer have been described for the following compounds:

1. Ag(I) and Cu(II) derivatives of 1,3-diaryltriazene-substituted sulfonamides [96];
2. Cu(II) agents with mixed 2-amino-2-thiazoline ligands [100];
3. Cu(II) complex derived from N,N′-bis (acetylacetone)-propylenediimine [107];
4. Pd(II) complexes containing a thiazoline derivative [69];
5. Ru(II) compounds with *N,O*-ligands [97];
6. Benzimidazole C,N-cyclometalatedarene Ru(II), Ir(III), and Rh(III) derivatives [99];
7. Diaminotris(phenolato) vanadium(V) complexes [108].

**Figure 8:**
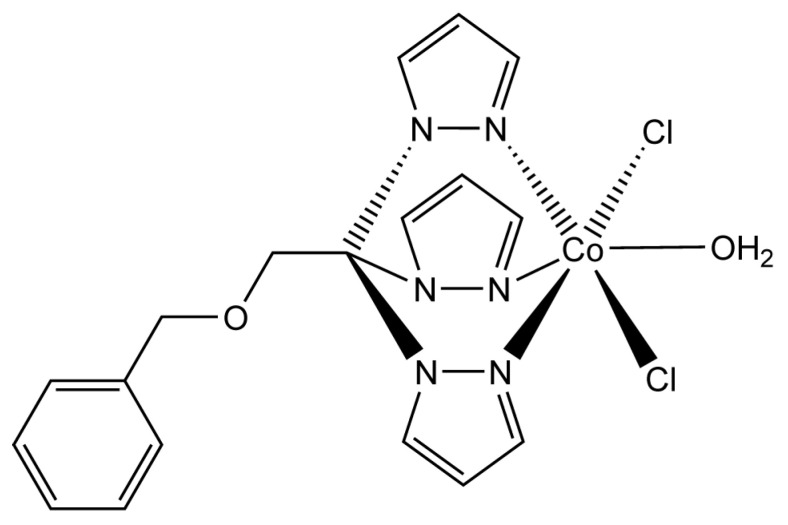
Chemical structure of cobalt scorpionate with tris(pyrazol-1-yl)methane ligands.

Mononuclear Co(II) and Zn(II) bis(3,5-di-t-butylpyrazol-1-yl)acetate compounds display antiproliferative properties against the colorectal carcinoma HCT 116 cell line [68]. The cytotoxic effects of cobalt scorpionates with tris(pyrazol-1-yl)methane ligands (Figure 8) towards HCT 116 cells are related to cell viability loss due to an increase in apoptosis [109].

The scorpionate molybdenum(IV) complexes hydrotris(3-isopropyl-1H-pyrazolyl) boro[2-methyl-3-(oxo-κO)-4*H*-pyran-4-thioato-κS]oxomolybdenum(IV) and hydrotris 3-isopropyl-1*H*-pyrazolyl)boro[6-methyl-3-(oxo-κO)-4*H*-pyran-4-thioato-κS]oxomolybdenum(IV) induce apoptosis in colorectal HCT 116 cells. These are the first cancerostatic molybdenum complexes bearing a trispyrazolylborate ligand that have been developed, and, in general, are the most cytotoxic molybdenum complexes [110].

**Figure 9:**
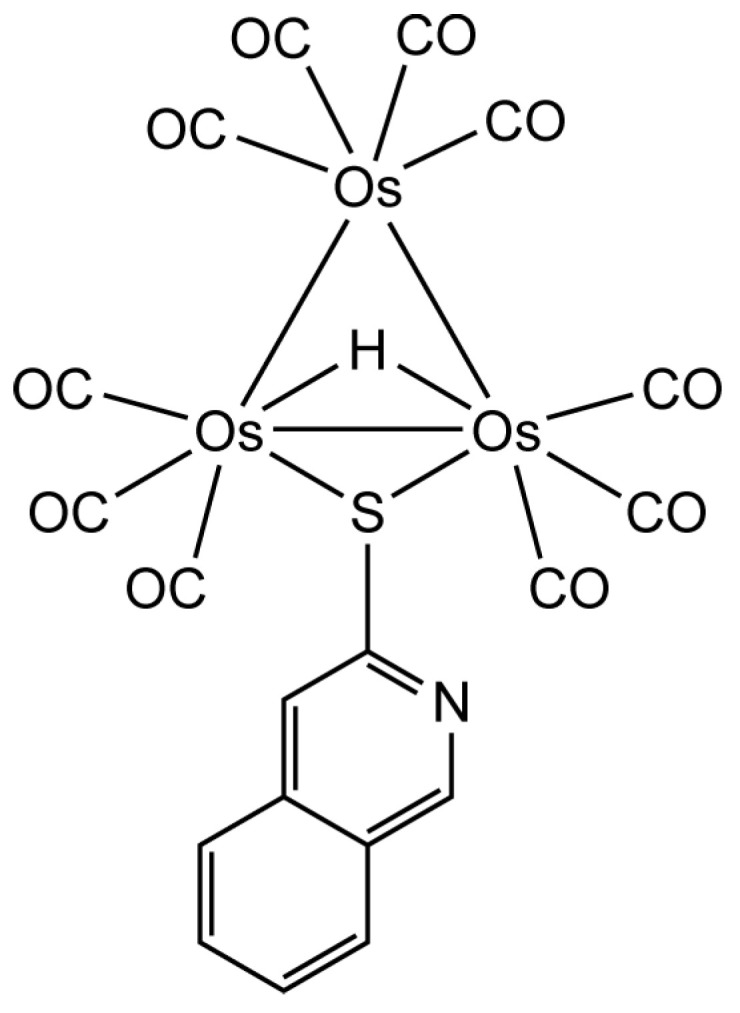
Chemical structure of a trisosmium carbonyl complex.

A trisosmium carbonyl complex (Figure 9) is cytotoxic against the colorectal cancer cell line HCT 116 due to the induction of apoptosis via mitochondrial stress and reactive oxygen species production, leading to a decrease in tumor growth rate and a 50% reduction in tumor volume. Combined therapy with Cisplatin and the trisosmium carbonyl complex has been shown to lead to synergistic effects [111].

**Figure 10:**
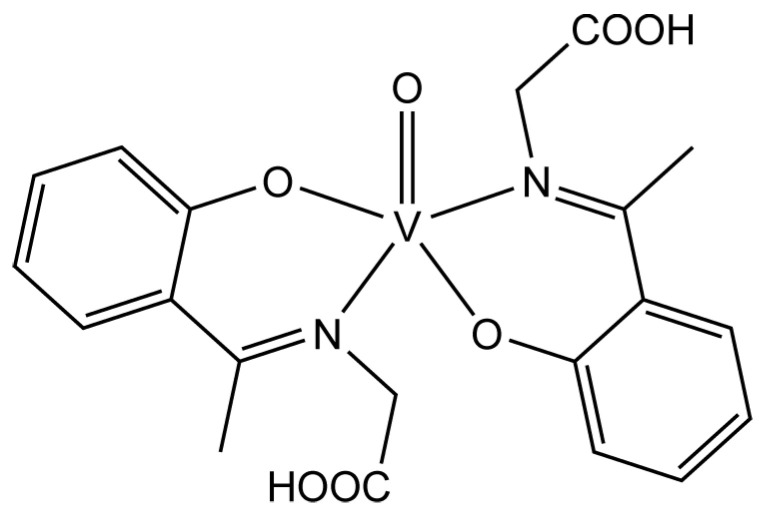
Chemical structure of vanadium complex with N-(2-hydroxyacetophenone) glycinate.

In the HCT116 cell line, the avanadium complex with N-(2-hydroxyacetophenone) glycinate (Figure 10) has been described to cause apoptosis through mitochondrial membrane permeabilization [112].

Palladium(II) complexes with 4-hydroxycoumarin bidentate ligands have been reported to exhibit antiproliferative effects against the HCT116 cell line [113].

### 5.6. Liver Cancer

Human hepatocellular carcinoma is the sixth most commonly diagnosed and the fourth leading cause of cancer-related deaths worldwide. For males, the disease ranks fifth in terms of global cases and second in terms of deaths, with both incidence and mortality rates three times higher than those in women. The disease is the seventh most common cancer in women [114].

The complex of Co(II) and 1,3,5-triazine-2,4,6-triamine hexaacetic acid ligand forms a metal–organic framework on the basis of multicarboxylate ligands and transition metal ions. It has been shown that this compound decreases the viability of liver cancer cells [115]. It has been reported that a Pd(II) complex with Schiff base derived from 4-Aminoantipyrine and 3-(hydroxyimino) butan-2-one exhibits cytotoxicity against the growth of human liver cancer cell lines [116].

The mechanism of copper-1,10-phenanthroline [117] and vanadium(V) terpyridine complex-induced apoptosis in hepatocarcinomam Bel-7402 cells [118] is associated with reactive oxygen species production, oxidative DNA damage [117] and decreased mitochondrial membrane potential [118].

A Ag(I) compound with a Miconazole ligand [119] and a Ag(I) derivative with a triazole-based N-heterocyclic carbene ligand (1-tert-butyl-4-(4-methylphenyl)-3-phenyl-1*H*-1,2,4-triazol-4-ium-5-ylidene) [120] both exert toxicity against the human hepatocellular carcinoma Hep G2 cell line through cellular membrane damage [119].

**Figure 11:**
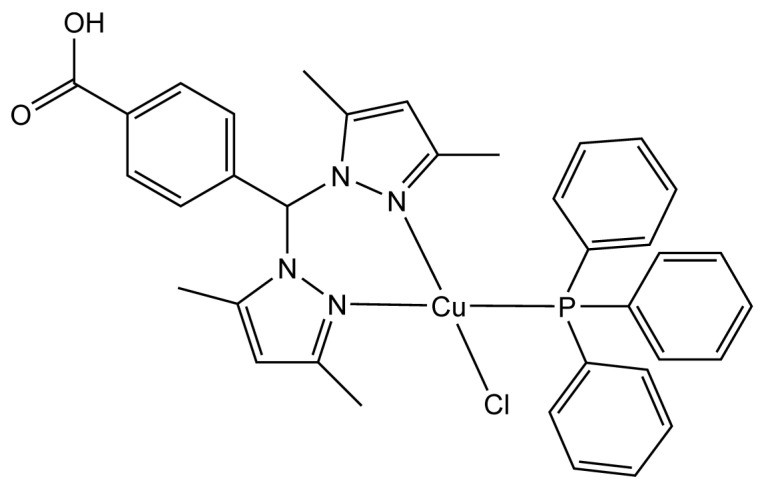
Chemical structure of copper(I) complex of “scorpionate” bis-pyrazolyl carboxylate ligand with auxiliary phosphine.

Through in vitro studies, a copper(I) complex of a “scorpionate” bis-pyrazolyl carboxylate ligand with auxiliary phosphine (Figure 11) has been found to exhibit cytotoxic activity against the growth of Hep G2 cells, and this effect is similar to that of Cisplatin [121].

Scorpionate cobalt with tris(pyrazol-1-yl)methane) ligands (Figure 8) has been reported to induce apoptosis in the Hep G2 cell line [109,122]. Vanadylbisacetylacetonate has also been described to block cell cycle progression past the G1 phase in the same cell line [123].

**Figure 12:**
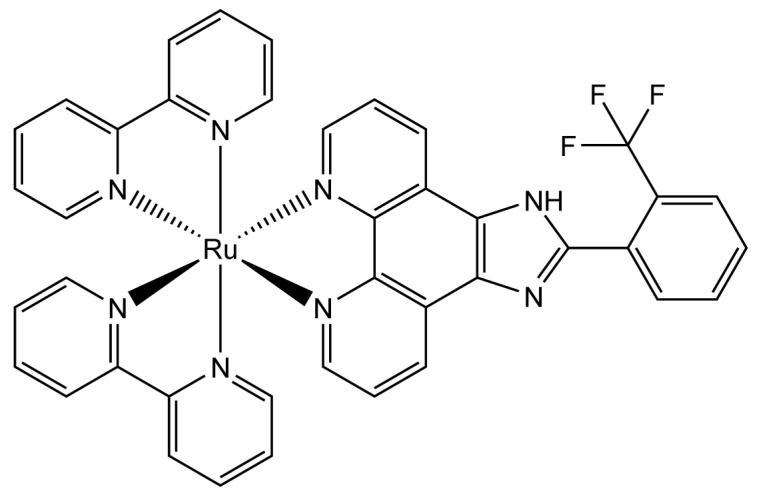
Chemical structure of Ru complex Λ-WH0402.

It has been demonstrated that the Ru complex L-WH0402 (Figure 12) induces HCCLM6 cell death by triggering Becl in-1-dependent autophagy pathways [124].

**Figure 13:**
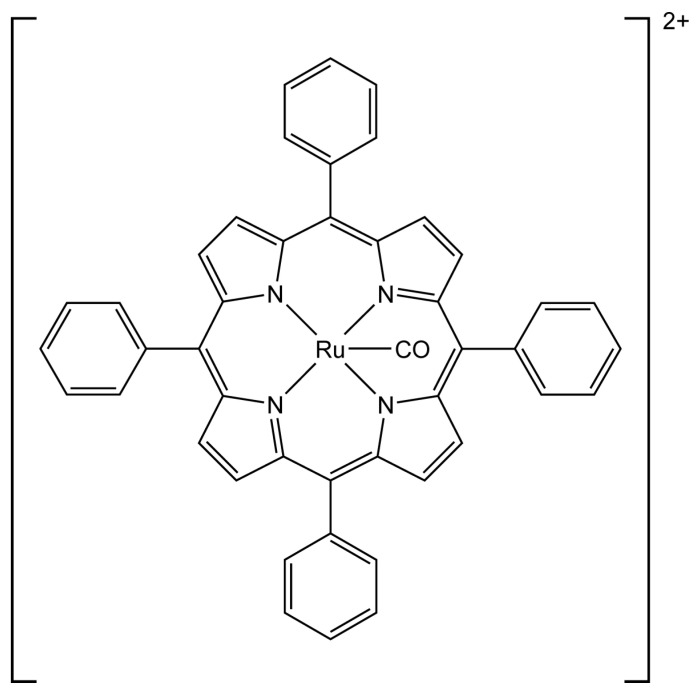
Chemical structure of Ru(II) carbonyl 5,10,15,20-tetraphenyl-21H,23H-porphine complex.

**Figure 14:**
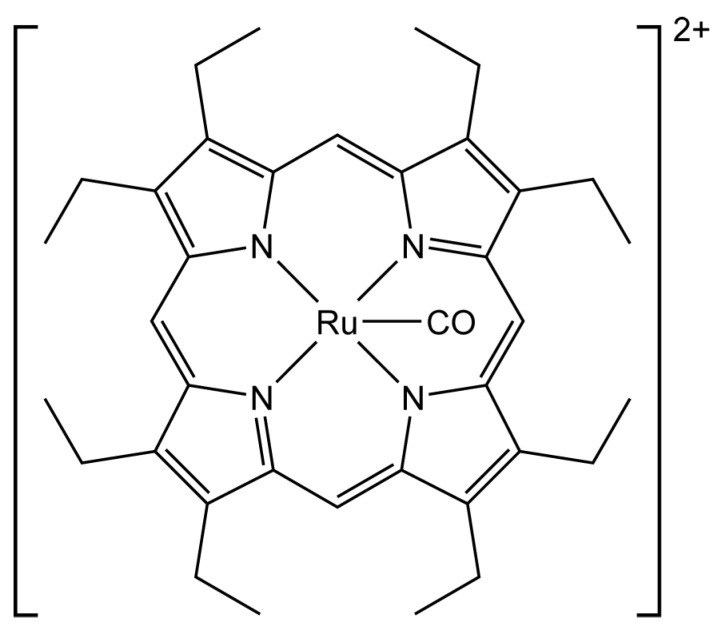
Chemical structure of Ru(II) carbonyl 2,3,7,8,12,13,17,18-octaethyl-21H,23H-porphinecomplex.

It has been described that the Ru(II) carbonyl 5,10,15,20-tetraphenyl-21H,23H-porphine complex (Figure 13) and Ru(II) carbonyl 2,3,7,8,12,13,17,18-Octaethyl-21H,23H-porphine complex (Figure 14) exert antiproliferative effects against the human hepatocellular carcinoma cell lines Bel7402, HepG2, and SMMC7721, and induce apoptosis and inhibit cell migration and invasion of HepG2 cells [125].

### 5.7. Pancreatic Adenocarcinoma

Pancreatic ductal adenocarcinomas are derived from the ductal epithelium of the pancreas and constitute 90% of pancreatic cancers [126,127].

It has been shown that oxidovanadium(IV) complexes with 2-methylnitrilotriacetate, N-(2-carbamoylethyl)iminodiacetate, and N-(phosphonomethyl)-iminodiacetate ligands are cytotoxic towards PANC-1 and MIA PaCa2 pancreatic cancer cells [113], but not towards hTERT-HPNE non-tumor human pancreas epithelial cells [128]. It has been demonstrated that, due to reactive oxygen species generation, bis(acetylacetonato)-oxidovanadium(IV) suppresses the proliferation of AsPC-1 human pancreatic cancer cells [129]. Palladium(II) complexes with 4-hydroxycoumarin bidentate ligands exert antiproliferative effects against MIA PaCa2 cancer cells [113].

### 5.8. Bladder Cancer

**Figure 15:**
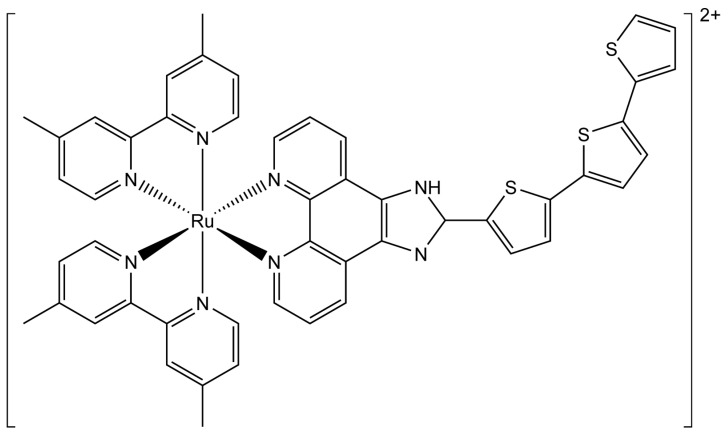
Chemical structure of Ru(II) polypyridyl complex TLD 1433.

Bladder cancer is the second most common malignancy of the urinary tract and is three times more common in men than in women [130]. Activity against bladder cancer has been proven for Ni(II) and Cu(II) complexes with N-arylalkyliminodiacetamides [131] and Ru(II) polypyridyl complex TLD 1433 (Figure 15) [83].

Copper(II) compounds with an N′-methylsarcosinamide/N,N′-dimethylglycinamide ligand exhibit cytotoxicity against the T24 and UM-UC-3 human bladder cancer cell lines [132]. It has been proven that polyoxometalate-based derivatives of Ag with 4-(5-(1H-imidazol-2-yl)-1H-1,2,4-triazol3-yl), and of Cu with 3-(pyrid-3/4-yl)-5(1H-1,2,4-triazol-3-yl)-1,2,4-triazolyl, exert anticancer effects on J82 bladder cancer cells. The quercetin–zinc agent blocks cell cycle progression in BFTC-905 human bladder cancer cells [95].

### 5.9. Ovarian Cancer

Ovarian cancer is the seventh most common cancer. Malignant epithelial ovarian neoplasms [133] are more common in North America and Europe than in Africa and Asia and result in abnormal cells that invade the lungs, liver, and abdomen [134]. Ovarian cancer is the most lethal gynecologic cancer and is the fifth leading cause of death in women in the US, with about 250,000 women diagnosed each year. Ovarian carcinoma is the most common type, which comprises 95% of cases and is divided into five main subtypes, of which high-grade serous carcinoma is the most common and germ cell tumors and sex cord-stromal tumors are rarer [135].

One serious problem in the treatment of ovarian cancer is the development of resistance to platinum-based chemotherapy. Alternative therapeutics for chemoresistant ovarian cancer include nanoparticles with metal–organic agents [136] and dinuclearmetal complexes [137].

It has been shown that the following compounds exert an antiproliferative effect on ovarian carcinoma cell line A2780cis:

1. Dinuclear Ag(I) pyrrolidine dithiocarbamato complex with a bidentate carbene ligand [137];
2. Pd(II)allyl agents with an N-trifluoromethyl ligand [138];
3. Mixed Cu(II)-phenanthroline derivatives [139];
4. Mononuclear Zn(II) and Co(II) 3,5-di-t-butylpyrazol-1-yl)acetate] complexes [68].

In ovarian cancer, cyclic amine and thioether metal complexes are shown to exert anticancer activity. Investigations have shown that Rh-1-aza-4.7-dithiacyclononane exerts antiproliferative effects on the ovarian cancer cell line NuTu-19 [140]. Apoptosis of ovarian cancer cells caused by the vanadium derivative oxidovanadium(IV), complexes of salicylaldimines, and polypyridyl ligands is deemed to be the result of induced changes in mitochondrial membrane potential [141].

It has been reported that in the ovarian carcinoma cell lines OVCAR-3, A2780, A2780cis, and A2780adr, the anticancer activity of diaminotris(phenolato) vanadium(V) complexes is based on the arrest of the cell cycle at the S phase [108].

### 5.10. Cervical Cancer

In terms of mortality and incidence, cervical cancer ranks fourth [142]. It has been found that the following metal-based complexes exhibit anticancer activity against the HeLa human cervical cancer cell line:

1. Au(I) complexes of an N-heterocyclic carbene ligand (1-tert-butyl-4-(4-methylphenyl)-3-phenyl-1H-1,2,4-triazol-4-ium-5-ylidene) [120];
2. Polyoxometalate-based compounds of Ag with 4-(5-(1H-imidazol-2-yl)-1H-1,2,4-triazol3-yl)pyridine) [95];
3. Ag(I) and Cu(II) derivatives of 1,3-diaryltriazene-substituted sulfonamides [95];
4. Cu(I) agents with cyclodiphosphazane and pyridyl ligands [90];
5. Cu(II) complex of the Schiff base ligand 3-(2-(1-(1H-benzimidazol-2-yl)ethylidene) hydrazinyl)quinoxalin-2(1H)-one [93];
6. Dinuclear Cu(II) compounds based on an N,N,O-chelating salphen-like ligand [92];
7. Cu(II) and Fe(II) derivatives with 8-hydroxyquinoline derivatives [143];
8. Ru(II) hydrazinyl-thiazoloarene agent [144].

Ru(II)hydrazinyl-thiazoloarene complexes [136] and benzimidazole C,N-cyclometalated Ru(II), Ir(III), and Rh(III) complexes exert cytotoxic effects against the *A2780* and A2780cisR cervical cancer cell lines [99].

**Figure 16:**
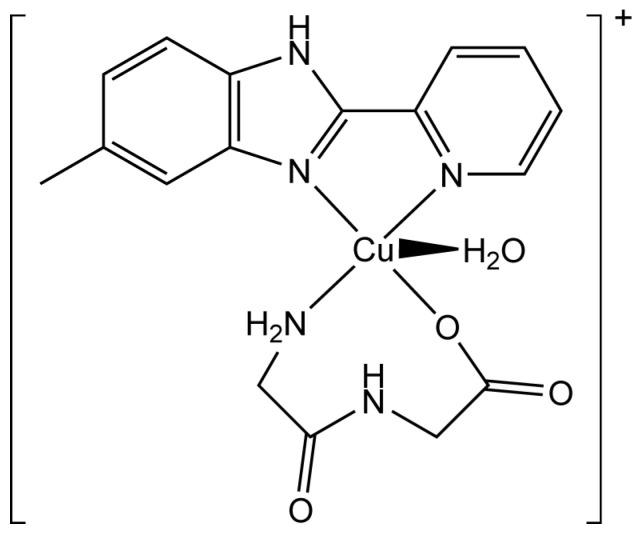
Chemical structure of Cu(II) complex of glycyl-glycine-5-methyl-2-pyridyl)benzimidazole.

It has been demonstrated that Cu(II) dipeptide complexes exhibit antiproliferative activity. Cu(II) complexes of glycyl-glycine-5-methyl-2-(2′-pyridyl) benzimidazole (Figure 16) have been reported to induce apoptosis in the A549 and HeLa cancer cell lines. Specifically, in HeLa cells, apoptosis has been observed to occur as a result of an ROS-mediated mitochondrial dysfunction pathway [145].

### 5.11. Testicular Cancer

The most common malignancy among young men is testicular cancer. The cytotoxic effects of metallocenes containing vanadium (vanadocene), titanium (titanocene), zirconium (zirconocene), molybdenum (molybdocene), and hafnium (hafnocene), linked to the organic ligands bis (cyclopentadienyl) or hexafluoroacetylacetonate by direct carbon-metal bonds, have been tested using the human testicular cancer cell lines Tera-2 and Ntera-2. It has been shown that, through inhibiting DNA synthesis, vanadium(IV)-containing metallocenes exhibit significant cytotoxicity against these cell lines [146].

### 5.12. Prostate Cancer

Prostate cancer is the second most common cancer in males, with 1.2 million diagnoses in this group in 2018. It is also the fifth leading cause of cancer-related death in men [147,148], with 359,000 deaths in the same year [142]. This cancer is more common in developed countries, where rates have been increasing [147].

**Figure 17:**
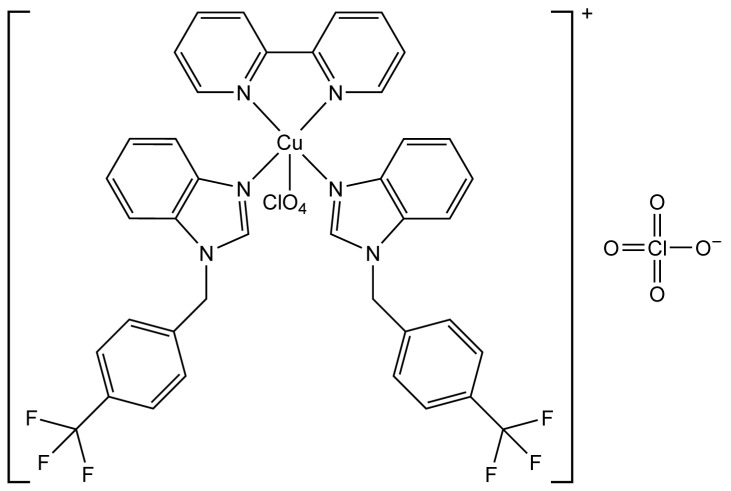
Chemical structure of copper(II) complex with 2,2′-bipyridyl and 1-(4-(trifluoromethyl) benzyl)benzyl)-1H-benzimidazole.

It has been found that metal-based complexes such as benzofuran-conjugated iridium [149] and Cu(II) complexes with 2,2′-bipyridyl and 1-(4-(trifluoromethyl)benzyl)-1H-benzimidazole ligands (Figure 17) [150] exhibit antiproliferative effects against the DU145 prostate cancer cell line.

Cu(II) complexes of glycyl-glycine-5-methyl-2-(2′-pyridyl)benzimidazole have been shown to induce apoptosis of the prostate cancer cell line PC-3. Furthermore, 1,3-diaryltriazene-substituted sulfonamides show in vitro cytotoxicity against the human prostate cancer cell lines DU-145 and PC-3 [96].

Schiff base copper derivatives of quinoline-2-carboxaldehyde have been reported to act as proteasome inhibitors of human LNCaP and PC-3 prostate cancer cells [151]. In vitro anticancer effects have been observed in the human prostatic cancer cell lines DU145 and PC-3 for the following substances:

1. Gold(III)-dithiocarbamato derivatives, which inhibit cell proliferation in a dose-dependent way, and are more active than Cisplatin [152];
2. Pd(II) compounds with a metal center coordinated with ligands such as curcumin and 4,4′-dinonyl-2,2′-bipyridine [153].

### 5.13. Leukemia

Leukemia represents a group of blood cancers that result in a high number of abnormal blood cells. Acute lymphoblastic leukemia is the most common type of leukemia in young children, while its chronic form mostly affects adults over the age of 55. Acute myelogenous leukemia occurs more commonly in adults than in children, and more often in men than in women. Subtypes of the disease include acute promyelocytic leukemia, acute myeloblastic leukemia, and acute megakaryoblastic leukemia [154].

Gold(I) derivatives of a triazole-based N-heterocyclic carbene (NHC) ligand (1-*tert*-butyl-4-(4-methylphenyl)-3-phenyl-1*H*-1,2,4-triazol-4-ium-5-ylidene) display activity against the leukemic cells CCRF-CEM and HL-60 [120]. Palladium(II) complexes with thiazoline ligands [155], such as 2-(2-pyridyl)imine-N-(2-thiazolin-2-yl)thiazolidine, promote cytotoxicity and exert pro-apoptotic effects against the human promyelocytic leukemia HL-60 cell line and lymphoma cell lines HL-60 and U-937, respectively [100].

Co(II), Cu(II), and Zn(II) complexes of the Schiff base ligand, 2,2′-(((((2-hydroxypropane-1,3-diyl)bis(oxy))bis(2,1-phenylene))bis(methylene))bis(bis(azanylylidene))bis(methanylylidene))bis(4-bromophenol) exhibit activity against the human leukemia cell line K562 in vitro [156].

**Figure 18:**
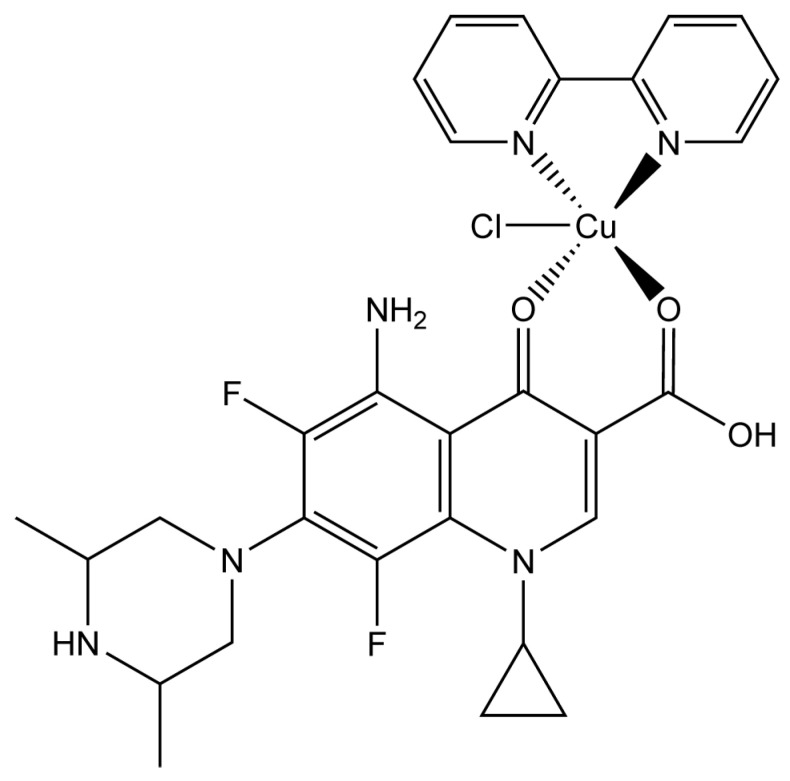
Chemical structure of copper(II) (Sparfloxacinato)(2,2-bipyridine)Cl complex.

**Figure 19:**
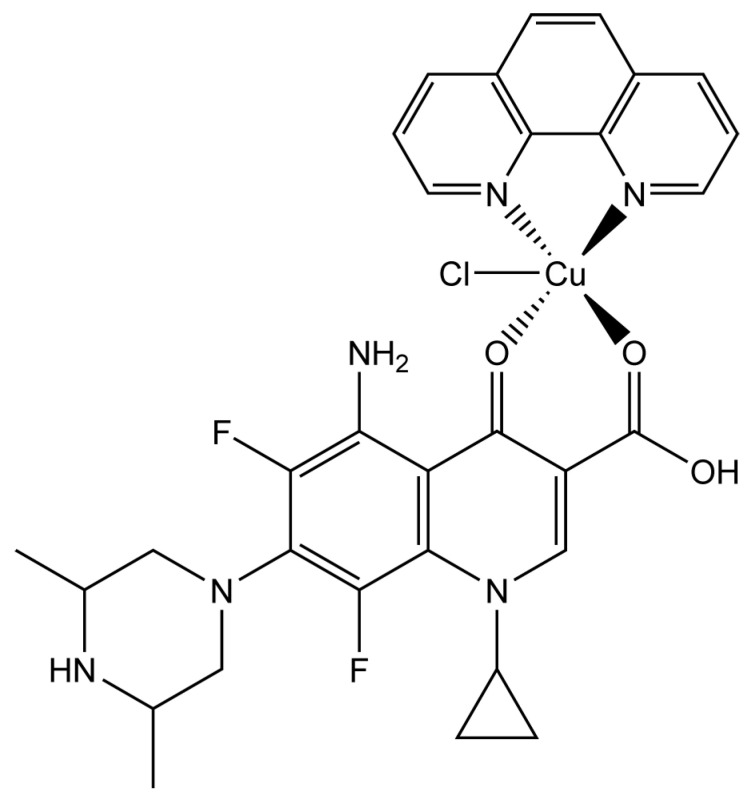
Chemical structure of copper(II) (Sparfloxacinato)(1,10-phenanthroline)Cl complex.

Copper(II) complexes with drugs are much more active in the presence of nitrogen-donor heterocyclic ligands, such as 2,2′-bipyridine and 1,10-phenanthroline. The Cu(Sparfloxacinato) (2,2-bipyridine)Cl (Figure 18) and Cu(Sparfloxacin) (1,10-phenanthroline)Cl (Figure 19) complexes have been reported to exhibit cytotoxic effects against the human leukemia cell line HL-60 (peripheral blood human promyelocytic leukemia) [157].

### 5.14. Sarcoma

Common types of sarcoma are osteosarcoma and chondrosarcoma [158]. It has been found that Co(II) coordination polymers containing 2,4,6-tri(1H-imidazol-1-yl)-1,3,5-triazine) suppress the growth of human osteogenic sarcoma MG-63 cells [159]. The aqua-(2,2′,2″-nitrilotriacetato)-oxo-vanadium compound exerts a cytotoxic effect against the human osteosarcoma MG-63 and HOS cell lines [160].

To increase the in vitro anticancer activity of Fluorouracil against human osteogenic sarcoma cells (MG63 and U2OS), a targeted anticancer drug delivery system has been developed based on an adenine/Zn(II)/4,4′-(aminomethylene)dibenzoic framework, which has pores into which Fluorouracil can be loaded through adsorption [161].

Ni(II) and Cu(II) coordination complexes with 2-(5-(pyridin-3-yl)-4-(pyridin-4-yl)-4H-1,2,4-triazol-3-yl)pyridine are proven in vitro antiproliferative agents against the human osteogenic sarcoma cell lines MG-63, U2OS, and SAOS-2 [162].

Moderate cytotoxicity against the human sarcoma cancer cells MES-SA and MES-SA/Dx5 has been demonstrated by Schiff base Cu(II) and Zn(II) complexes with the following ligands [163]:

1. 2-[N-(1H-benzimidazol-2-ylmethyl)ethanimidoyl]aniline;
2. N-[1-(pyridin-2-yl)ethylidene]-N′-(2-{[1-(pyridin-2-yl)ethylidene]amino}ethyl);
3. Propane-1,3-diamine;
4. 2-(pyridin-2-yl)-N-[1-(pyridin-2-yl)ethylidene]ethanamine.

It has been reported that *cis*-[RuCl(benzonitrile)(1,10-phenanthroline) (4-bis-(diphenylphosphino)butane)]PF_6_ is a more potent inhibitor of sarcoma-180 tumor cell viability than of normal lymphocytes, the mechanism of which involves suppression of e G0/G1 phase in cancer cells [164].

### 5.15. Mesothelioma, Neuroblastoma, and Brain Glioma

Mesothelioma is a type of cancer that originates from the thin layer of tissue that covers a significant number of the interior organs, most often affecting the pleura covering the lungs and chest. More than 80% of mesothelioma cases are caused by exposure to asbestos [165].

A copper(II) complex with ligands 2,2′-bipyridyl and 1-(4-(trifluoromethyl) benzyl)benzyl)-1H-benzimidazole has been found to exert antiproliferative effects against the SPC212 mesothelioma cell line [150].

There are more than 120 different types of brain tumors, the more common being neuroblastoma, glioblastoma, medulloblastoma, and diffuse midline glioma. Most brain tumors, such as a glioblastoma multiforme, are malignant and potentially fast-developing. Neuroblastomas are cancers that originate from the undifferentiated cells (neuroblasts) of the sympathetic nervous system [166].

Oxindole–Schiff base Cu(II) compounds exhibit potential cytotoxic effects against human neuroblastoma SH-SY5Y cells [167].

A glioma is a kind of tumor that originates from the glial cells of the brain or spine, and comprises about 30% of all central nervous system tumors and 80% of all malignant brain tumors [166].

Cationic N-heterocyclic carbine Cu(I) complexes coordinated to 2,2-bis-pyridyl ligands exert cytotoxic activity against the human brain glioma HS-683 cell line [77].

Vanadium complexes with N-(2-hydroxyacetophenone) glycinate are active against U-373MG glioblastoma cells and trigger apoptosis through mitochondrial membrane permeabilization [108].

### 5.16. Melanoma

**Figure 20:**
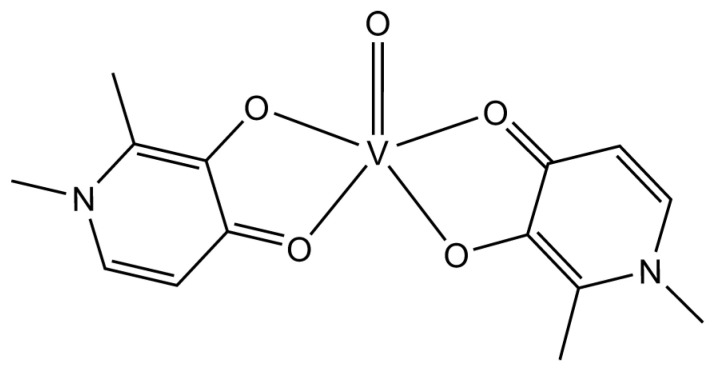
Chemical structure of pyridone-based vanadium complex.

Melanoma is the most aggressive skin cancer [168] and is responsible for most cutaneous malignancy-related deaths. The antitumor effects of vanadium compounds against malignant melanoma cell lines are a result of cell cycle arrest occurring at different phases. In malignant melanoma cell lines, the antitumoral effect of the pyridone-based vanadium complex (Figure 20) is a result of apoptosis induction [169].

It has been reported that Cu(II) compounds with ligands such as 1.10-phenanthroline [93], benzimidazole [93], aziridine [151], quinoline [151], and flavonoids [170] exert antiproliferative effects against different melanoma cell lines.

The following compounds have been described to exhibit anticancer activity towards B16-F10 melanoma cells:

1. Dinuclear Cu(II) phenanthroline μ-azido complex and Cu(II) agent with Schiff base ligand [93];
2. Cu(II) derivatives of 3-(2-(1-(1H-benzimidazol-2-yl) ethylidene)hydrazinyl) quinoxalin-2(1H)-one, synthesized through the condensation of 3-hydrazinylquinoxalin-2(1H)-one and 1-(1H-benzoimidazol-2-yl) ethanone [93];
3. Schiff base copper derivatives of quinoline-2-carboxaldehyde [151].

Copper(II) complexes of aziridine ligands exhibit potential cytotoxic effects against WM-115 melanoma cells [151]. Copper(II) derivatives with flavonoid ligands of chrysin, apigenin, and genistein also exert anticancer effects against 518A2 melanoma cells [170].

Auranofin with Au(I) thiocarbamoyl–pyrazoline ligand complexes also exerts anticancer effects. Dinuclear Pd(II) derivatives can suppress A2058, B16-F10-nex2, ME1402, SK-MEL-5, SK-MEL-110, WM1158, and 501mel melanoma cells, and palladium(II) allyl complexes with N-trifluoromethyl ligand exert antiproliferative effects against the A375 malignant melanoma cell line [140]. Human skin malignant melanoma SK-Mel-28 cells have been observed to be inhibited by cationic N-heterocyclic carbene Cu(I) complexes coordinated to 2,2′-bis-pyridyl ligands [77], dinuclear Pd(II) compounds [160], and Ru(II) complexes of 2-(pyren-1-yl)-1*H*-imidazo[4,5-*f*] [1,10] phenanthroline] ligands [171].

The antiproliferative activity of scorpionate silver(I) tris(pyrazolyl)methanesulfonate and silver(I) 1,3,5-triaza-7-phosphadamantane complexes against the A375 human malignant melanoma cancer cell line is caused by DNA interactions [172].

## 6. Effect of Heavy Metals on the Body and Cancer After Release from Metal-Based Complexes

After the application of organometal complexes, they can undergo hydrolysis or ligand exchange and release their metal ions. These free metal ions can inhibit the function of enzymes or damage DNA by binding to thiol, amino, and phosphate groups in both structures. Metal ions like Fe^2+^ [173], Cu^+^ [174], or Ni^2+^ released after reacting with molecular oxygen can generate superoxide (O_2_^•−^), which destroys proteins and lipids. On one hand, this is a mechanism through which cancer cells can be damaged; on the other hand, this can also lead to high toxicity against normal cells [173].

An important research trend is the development of compounds with high selectivity for cancer cells and low toxicity against healthy cells. Some ruthenium compounds, such as organoruthenium(II) complexes, are prospective anticancer agents [175] due their selective toxicity toward cancer cells compared to normal cells [176].

Ferrocene derivatives can release Fe^2+^, which induces oxidative stress by generating reactive oxygen species. This mechanism can induce oxidative stress and lead to cell apoptosis. Some ferrocene derivatives may cause greater ROS formation in cancer cells compared to normal cells, which would be advantageous for cancer therapy as it could specifically suppress the growth of Mn(II) cells with oncogenic mutations like those in K-Ras proteins in pancreatic, colorectal, and lung cancers [173].

Copper-induced oxidative stress leads to a type of cell death called cuproptosis [174], which is exploited as a therapeutic mechanism in lung cancer [177] as well ashepatocellular carcinoma [178].

One of the body’s mechanisms for reducing toxicity includes the binding of metal ions by proteins such as metallothioneins. Thanks to its ability to neutralize reactive oxygen species and bind metals, glutathione functions as an antioxidant. Other detoxification mechanisms include the biliary and renal excretion pathways [179].

## 7. Comparison of Effects of Anticancer Metal-Based Compounds and Cisplatin

The disadvantages of Cisplatin in anticancer treatment are connected to its poor selectivity and toxicity against normal organs, and the occurrence of resistance in cancers to its effects [180]. An important goal in anticancer treatment development is the creation of non-platinum metal–organic complexes with proven advantages over Cisplatin, including higher antiproliferative effects, better selectivity, decreased toxicity, and the ability to prevent resistance in cancer.

It has been reported that Cu(I) iodide complexes with aminomethyl(diphenyl)phosphine Lomefloxacin exert better activity (IC_50_ = 18 μM) than Cisplatin (IC_50_ = 31 μM) against MCF7 human breast adenocarcinoma [181].

The advantages of ferrocene derivatives, compared to Cisplatin, are their superior activity and lower toxicity. Ferrocene–quinoline hybrids (IC_50_ = 310 nM) exhibit higher activity than Cisplatin (IC_50_ = 13 μM) against WHCO1 esophageal cancer cells [182].

In comparison with Cisplatin, ruthenium complex NAMI-A, which contains imidazole and dimethylsulfoxide ligand, has low toxicity [183].

The resistance of tumor cells to Cisplatin is a result of lower cell uptake and enhanced efflux of the drug, thus leading to low intracellular concentrations. To overcome Cisplatin resistance in cancer cells, metal complexes with copper, gold, iron, ruthenium, and palladium have been developed. The potential mechanism of the antiproliferative effects of these metal complexes against Cisplatin-resistant cancers involves targeting the mitochondria or inhibiting proteasomes [179].

Various copper-based compounds have been tested on colon, breast, prostate, and ovarian cancer cells, demonstrating their ability to induce the activity of pro-apoptotic molecules such as p53, BAX, caspase-8, and caspase-9. The effects of copper complexes, Au(III) dithiocarbamates, and Au(III) polypyridyl complexes are based on the inhibition of thioredoxin reductase, inducing apoptosis of both sensitive and resistant ovarian cancer cells through oxidative stress generation [179]. Ferrocenyl–benzimidazole hybrids (IC_50_ = 80 nM) possess stronger activity against Cisplatin-resistant cancer cells than Cisplatin (IC_50_ = 90 μM) [184].

Currently, there are clinical trials investigating a Ru(III) complex named BOLD-100 (NKP-1339), which exhibits greater potential than platinum drugs, due to its ability to target the endoplasmic reticulum. Ru(II)-cyclometalated complexes reduce glutathione levels and increase oxidative stress in cancer. The antimetastatic effect of organometallic Ru(II) compounds on resistant tumors is due to their interaction with proteins and ability to disrupt protein homeostasis in cancer cells. The increased selectivity of Ru(II) complexes for cancer cells is the reason for their superior antiproliferative activity compared to that of Cisplatin. The activity of Pd(II) complexes with thioamide ligands against resistant cells is due to their ability to induce mitochondrial dysfunction and apoptosis [179].

An important strategy in anticancer treatment development is the synthesis and clinical investigation of non-platinum metal complexes with superior anticancer activity and improved selectivity compared to Cisplatin, combined with lower toxicity and side effects, as well as the ability to avoid resistance.

## 8. Conclusions

In this review, we summarized the potential benefits of using non-platinum metal-based organic complexes to target different cancer cell types such as lung, gastric, colon, colorectal, liver, pancreatic, cervical, testicular, and prostate cancers, leukemia, sarcoma, mesothelioma, neuroblastoma, glioma, and melanoma. These metal-based agents have been reported to hold potential as anticancer drugs as they exhibit antiproliferative activity against different cancer cell types: lung [Cd(II), Co(II),Cu(II), Ni(II), Ru(II), Zn(II)]; breast (Ag(I), Cu(I), Cu(II), Ir(III), Ni(II), Rh(III), Ru(II)]; gastric [Cu(II), Cu(II)-La(III)]; colon [Ag(I), Cu(II), Ir(III), Pd(II), R(III), Ru(II), vanadium(V)]; colorectal [Ag(I), Co(II), Cu(II), Zn(II)]; liver [Ag(I), Co(II), Cu(II),Gd(III), vanadium(V)]; pancreatic [vanadium(IV)]; bladder [Ag(I), Cu(II), Ru(II)]; cervical [Ag(I), Au(I),Cu(I), Cu(II),Fe(II), Ir(III), Rh(III), Ru(II)]; testicular [vanadium(IV)]; prostate [Cu(II), Mn(II), Pd(II), Zn(II)]; leukemia [Ag(I), Co(II), Cu(II), Pd(II), Zn(II)]; sarcoma [Co(II), Ni(II), Zn(II)]; mesothelioma [Cu(II)]; neuroblastoma [Cu(II)]; brain glioma [Cu(II)] Mn(II); and melanoma [Au(I), Cu(II), Pd(II), Ru(II)]. The synthesis and clinical investigation of non-platinum metal complexes of copper, gold, iron, ruthenium, and palladium—which have superior anticancer effects and improved selectivity compared to Cisplatin, combined with lower toxicity and side effects, as well as the ability to avoid resistance—represent an important trend in anticancer treatment development.

## Figures and Tables

**Table 2:** Physical, chemical, and biological risk factors for the development of cancer.

Physical Factor	Physical Factor Effects	Type of Cancer
Ionized radiation	Gene mutation or chromosome aberration	Lung and thyroid cancer [17]
UV-light from solar radiation	DNA damage	Melanoma [18]
**Chemical factor**	**Chemical factor effects**	**Type of cancer**
Benzo-pyrene from tobacco	Mutation in the tumor suppressor gene p53	Lung cancer [19]
Ethyl alcohol	Mutation in the tumor suppressor gene p53	Esophageal cancer [20]
Cadmium	Gene mutations	Prostate cancer [21]
Aspergillus flavus metabolite Aflatoxin B1 (AFB1)	Gene mutations; DNA adduct formation	Liver cancer [22]
Heterocyclic amines from overcooked meat and fish [23]	DNA adducts, leading to mutations	Colon, breast, and pancreatic cancer [16]
**Biological factor**	**Biological factor effects**	**Type of cancer**
Helicobacter pylori infection	Inflammation, DNA damage, uncontrolled cell growth [24]	Gastric cancer [25]
Hepatitis B virus or Hepatitis C virus	Chronic inflammation, oxidative stress, disruption of tumor suppressors, mutations	Hepatocellular carcinoma [26]
Human T-lymphotropic virus type 1	DNA damage	T cell lymphoma [27]
Human papillomavirus	DNA damage	Esophageal cancer [28]

**Table 3:** Anticancer mechanisms of Auranofin.

	Cancer Type	Anticancer Mechanism of Auranofin
1	Calu-6, A549, SK-LU-1, NCI-H460, and NCI-H1299 lung cancer cells	Decrease in glutathione [35] Enhanced formation of reactive oxygen species [35] Decreased membrane potential in the mitochondrial membrane, resulting in a decrease in anti-apoptotic proteins [34]
2	Cisplatin-resistant ovarian cancer cell line OV2008/C13 Glio- and neuroblastoma brain tumors Head and neck squamous carcinoma	Inhibition of thioredoxin reductase [36]
3	HepG2 liver hepatocellular carcinoma cells MCF-7 cells breast cancer cells Chronic myeloid leukemia (CML)	Suppression of tumor growth Inhibition of proteasome-associated deubiquitinases [36,38]
4	Mesothelioma cells	Increased ROS levels; translocation of apoptosis-inducing factor (AIF) into the nucleus for activation of caspase-independent cell death [34]
5	Human ovarian carcinoma SKOV-3 cells	Caspase-3-mediated apoptosis [34]
6	Human pancreatic adenocarcinoma Non-small-cell lung cancer	Inhibition of the phosphatidylinositol 3-kinase/Akt, a regulator of cell survival under stress [34] Blockage of mammalian target of rapamycin (mTOR), a serine/threonine kinase important for protein synthesis [34]

**Table 4:** Metals used in anticancer metal-based complexes.

Metals			
Ag(I) [42] Au(I) [42] Au(III) [43,44] Ce(III) [45] Co(II) [46] Co(III) [47]	Cr [48] Cu(II) [49] Fe(II) [50] Fe(III) [51] Mo(II) [52] Ni(II) [53]	Zn(II) [54] Eu(III) [55] La(III) [55] Ir(III) [56,57] Nd(III) [45] Os(II) [56,57]	Pd(II) [58] Re(I) [56] Rh(III) [57,59] Ru(II) [57,60] Ti [61] Ru(III) [62] Ti(IV) [62] V(IV) [63]

## Data Availability

No new data were created or analyzed in this study. Data sharing is not applicable to this article.

## Abbreviations

AKT	Protein Kinase B
CLL	Chronic Lymphocytic Leukemia
DNA	Deoxyribonucleic acid
DUBs	Deubiquitinases
ERK	Extracellular Signal-Regulated Kinase
FDA	Food and Drug Administration
GSH	Glutathione
MAPK	Mitogen-Activated Protein Kinase
PI3K	Phosphoinositide 3-kinase
ROS	Reactive oxygen species
T-ALL	T-cell Acute Lymphoblastic Leukemia
